# Mutation of Ser172 in Yeast β Tubulin Induces Defects in Microtubule Dynamics and Cell Division

**DOI:** 10.1371/journal.pone.0013553

**Published:** 2010-10-21

**Authors:** Fabrice Caudron, Eric Denarier, Jenny-Constanza Thibout-Quintana, Jacques Brocard, Annie Andrieux, Anne Fourest-Lieuvin

**Affiliations:** 1 Grenoble Institut des Neurosciences, Institut National de la Santé et de la Recherche Médicale Unité 836, Université Joseph Fourier – Grenoble 1, Grenoble, France; 2 Groupe Physiopathologie du Cytosquelette, Institut de Recherches en Technologies et Sciences pour le Vivant, Direction des Sciences du Vivant, Commissariat à l'Energie Atomique et aux Energies Alternatives, Grenoble, France; 3 Institute of Biochemistry, ETH Zurich, Zurich, Switzerland; 4 Centre Hospitalier Lyon Sud, Pierre-Bénite, France; CNRS UMR6543, Université de Nice, Sophia Antipolis, France

## Abstract

Ser172 of β tubulin is an important residue that is mutated in a human brain disease and phosphorylated by the cyclin-dependent kinase Cdk1 in mammalian cells. To examine the role of this residue, we used the yeast *S. cerevisiae* as a model and produced two different mutations (S172A and S172E) of the conserved Ser172 in the yeast β tubulin Tub2p. The two mutants showed impaired cell growth on benomyl-containing medium and at cold temperatures, altered microtubule (MT) dynamics, and altered nucleus positioning and segregation. When cytoplasmic MT effectors Dyn1p or Kar9p were deleted in S172A and S172E mutants, cells were viable but presented increased ploidy. Furthermore, the two β tubulin mutations exhibited synthetic lethal interactions with Bik1p, Bim1p or Kar3p, which are effectors of cytoplasmic and spindle MTs. In the absence of Mad2p-dependent spindle checkpoint, both mutations are deleterious. These findings show the importance of Ser172 for the correct function of both cytoplasmic and spindle MTs and for normal cell division.

## Introduction

In mammalian cells, as in the budding yeast *Saccharomyces cerevisiae*, microtubules (MTs) form a dynamic network essential for many cellular processes, including cell polarity, organelle positioning and cell division. During mitosis, the interphase MT network rearranges to form a mitotic spindle responsible for chromosome segregation between daughter cells. In mammalian cells, during prophase and metaphase the nuclear envelope breaks down while MTs contact kinetochores to form the spindle. Budding yeast undergo a ‘closed mitosis’ during which the nuclear envelope does not break down while MTs form an intranuclear spindle [1]. Astral MTs, also called cytoplasmic MTs (cMTs), are anchored by their minus ends on the cytoplasmic face of the spindle pole bodies (SPBs) while their plus ends are oriented towards the cell cortex.

Contacts between the cMTs and the cell cortex are primordial for a correct positioning of the yeast mitotic nucleus at the neck between mother and bud cells. This positioning of the nucleus will eventually result in an accurate segregation of the genetic material between the two cells [2], [3], [4]. In mammalian cells, astral MTs of the mitotic spindle are also required to position the metaphase plate where the cleavage furrow will take place [5], [6], [7].

Proper interactions between MTs and the cell cortex rely on MT dynamic properties. MTs in all eukaryotic cells exhibit a dynamic instability behavior, plus ends of MTs alternatively undergoing phases of growth, shrinkage, and pause [8]. Transitions from growth to shrinkage are called ‘catastrophes’, while transitions from shrinkage to growth are called ‘rescues’. Intrinsic dynamic properties of MTs are highly controlled *in vivo* by a plethora of effectors [9], [10], [11], [12]. Among these effectors, plus-end tracking proteins (+TIPs) specifically accumulate at microtubule plus ends and are conserved in all eukaryotes [7], [12], [13].

In the budding yeast, correct positioning of the nucleus during mitosis depends on two independent genetic pathways involving several +TIPs. One spindle positioning pathway, called the Kar9p pathway, is active during metaphase and involves Kar9p, Bim1p (which is related to EB1) and Myo2p (type V myosin) [2], [14], [15], [16]. The other spindle positioning pathway, the dynein pathway, acts at anaphase onset and involves Bik1p (homologous to CLIP170), the kinesin-related Kip2p and the dynein heavy chain Dyn1p [17], [18]. One spindle positioning pathway can rescue the other, but inactivation of both Kar9p and dynein pathways impairs nuclear segregation and is lethal [14]. Likewise, in mammalian cells, spindle positioning depends on +TIP-mediated interactions of astral MTs with the cell cortex [5], [6], [7].

The building block of MTs, the tubulin dimer, is subjected to post-translational modifications such as acetylation, detyrosination or phosphorylation. While there is little evidence for a direct role of these post-translational modifications in the regulation of MT dynamics, it seems now clear that these modifications mark subpopulations of MTs and selectively affect downstream MT-based functions [19]. In yeast for instance, we showed that removal of the C-terminal aromatic residue of α tubulin disabled the interaction of Bik1p with plus ends of MTs [20]. Failure of Bik1p interaction with MT plus ends impaired spindle positioning at bud neck and affected yeast mitosis. These experiments outlined that the state of tubulin has profound consequences *in vivo*.

In this context, we found in mammalian cells that Cdk1, a key enzyme for cell division, is able to directly phosphorylate β tubulin on the Serine 172 residue [21]. Besides, this Ser172 residue has been found mutated in a human neuronal pathology called asymmetrical polymicrogyria, stressing the importance of this amino acid in the normal function/conformation of β tubulin [22].

Therefore, to get insights into the function of Ser172 in β tubulin, we constructed *S. cerevisiae* yeast strains mutated on Ser172 in Tub2p. In mutant cells, mitosis was impaired and MT dynamics were modified, with evidences for an abnormal function of +TIPs. These results indicate that this site in β tubulin is crucial for normal MT dynamics and cell division.

## Results

### Yeast as a cell model for the study of Ser172 in β tubulin

Ser172 of β tubulin is an important residue that is mutated in a human brain disease and phosphorylated by the cyclin-dependent kinase Cdk1 in mammalian cells. In order to detect whether yeast β tubulin was phosphorylated on Ser172 as in mammals, we purified yeast tubulin and analyzed it by HPLC-MS/MS. No phospho-Ser172 peptide was detected using this technique (data not shown). However, Ser172 phosphorylation in yeast is still conceivable because even in mammals the phosphorylated tubulin represents less than 1% of total β tubulin [21]. Thus, we have used yeast and its unique *TUB2* gene, as a model system to examine the cellular consequences of Ser172 modification.

**Figure 1:**
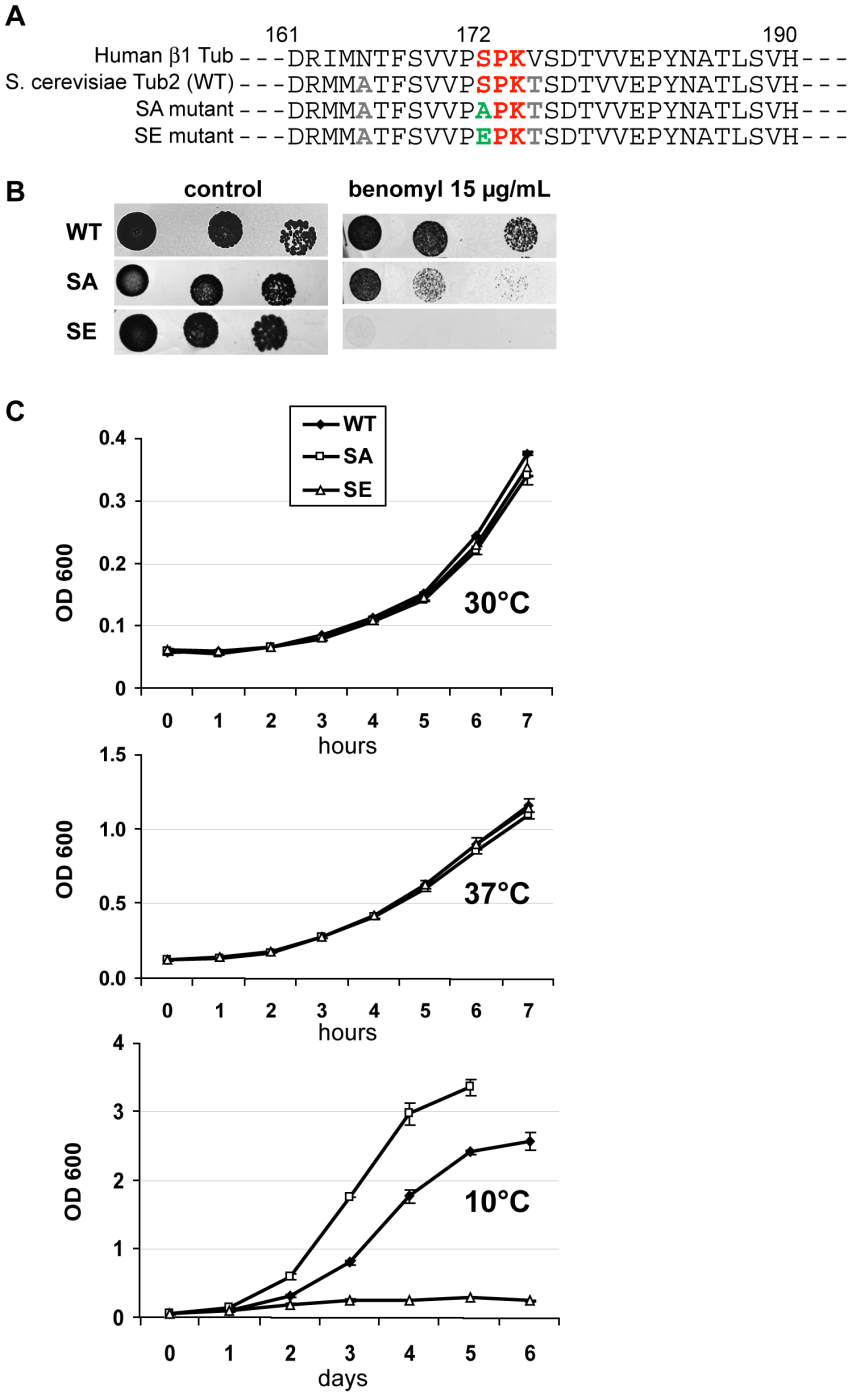
SA and SE cells are benomyl-supersensitive; SE cells are cold-sensitive. (A) Partial protein sequence of *S. cerevisiae* Tub2p (WT), and Tub2p with S172A or S172E mutations (SA or SE mutants), aligned with human β1 tubulin sequence. (B) Growth at 30°C of sequential dilutions of WT, SA and SE cells spotted on YPD media without benomyl (control) or containing 15 µg/ml of benomyl. (C) Measurement of optical density (OD) at 600 nm of cultures of 2 clones of WT, SA and SE cells in liquid YPD during several hours at 30°C or 37°C, or during several days at 10°C. Values are mean ± SEM.

We constructed a *tub2* mutant in which Ser172 was replaced by a neutral Alanine, the *tub2*-S172A or SA mutant, and a mutant in which Ser172 was replaced by a Glu residue to mimic phosphorylation, the *tub2*-S172E or SE mutant. The strategy used to obtain such mutants was as described in [23]. The aim of the strategy was to insert a mutant *tub2* gene copy (either SA or SE) into its normal locus (see Materials and Methods, and Figure 1A for partial amino-acid sequence of mutants). Wild type *TUB2* cells (WT cells) used in this study were constructed the same way, so that mutant and WT cells share a common genotype except for *TUB2* sequences.

### SA and SE strains are viable but exhibit benomyl supersensitivity and growth defects

Both SA and SE mutant haploid cells were viable in normal growth conditions (see Figure 1C, 30°C).

Supersensitivity to the microtubule depolymerizing drug benomyl is often observed in tubulin mutants [23]. Sensitivity of WT, SA and SE cells to benomyl was tested by spotting serial dilutions of cells either on normal YPD plates (control) or on YPD plates containing benomyl, and examining cell growth at 30°C (Figure 1B). Compared to WT cells, growth of SA and SE mutant cells was impaired on benomyl-containing medium, showing that both mutants were benomyl-supersensitive, SE even more than the SA mutant.

The supersensitivity to benomyl could be concomitant with cold-sensitivity [23]. Therefore, we examined cell growth of WT, SA and SE cells at 30°C, 37°C or 10°C (Figure 1C). At 30°C and 37°C, the three strains exhibited similar growth. At 10°C however, there were strong differences between strains: SA strain grew better than WT strain while SE strain growth was dramatically impaired (Figure 1C, 10°C). Thus, in SE strain, benomyl supersensitivity was associated with cold sensitivity, but it was not the case for SA strain. Such differences between SA and SE strains at 10°C could reveal differences in microtubule properties and in defects in the cell cycle (see below).

### Defects in mitotic phase of the cell cycle

**Figure 2:**
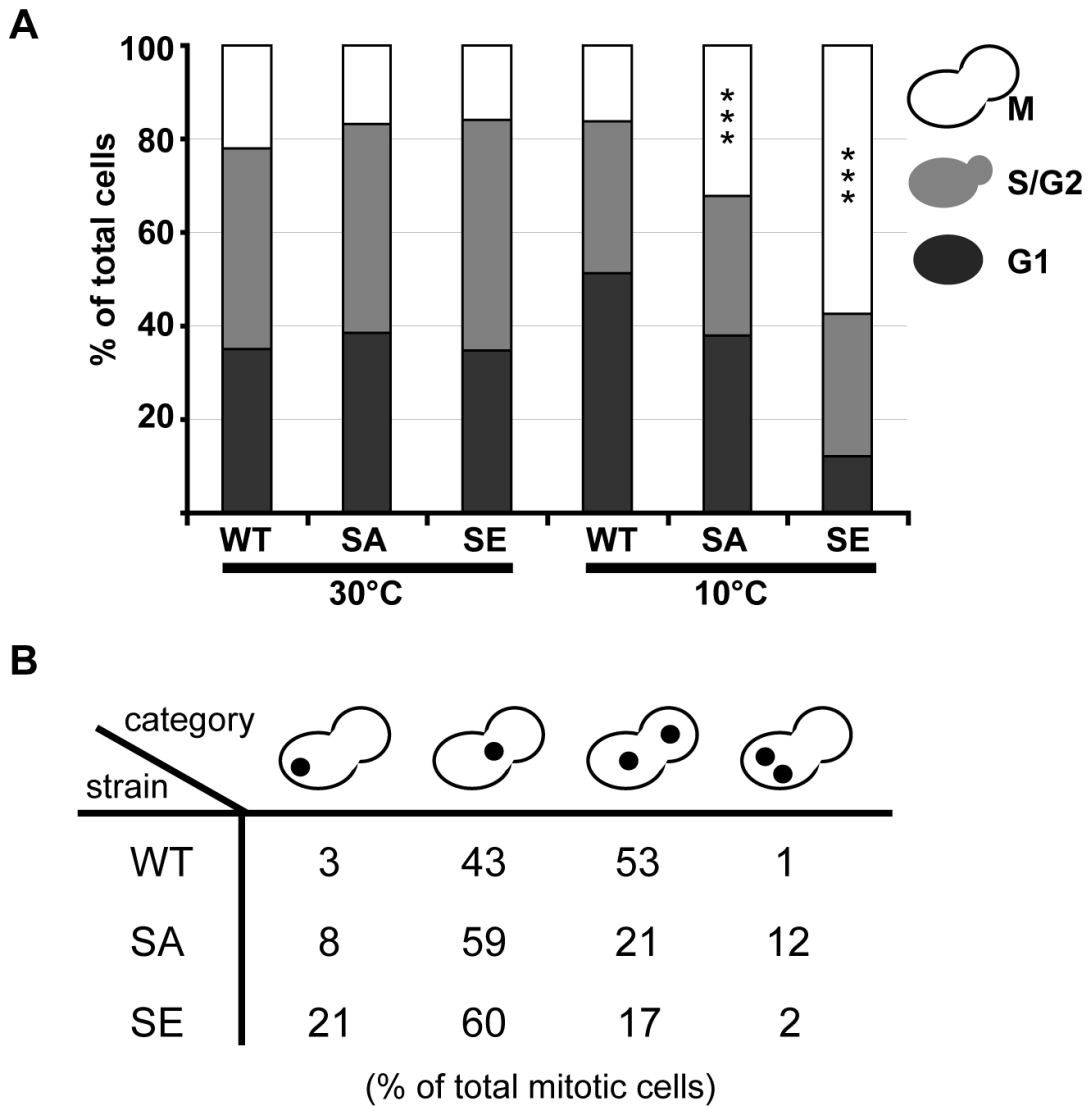
At the restrictive temperature of 10°C, SE cells undergo a mitotic block, and nuclei are mis-located and mis-segregated in mitotic SA and SE cells. (A, B) WT, SA, and SE diploid cells grown overnight in liquid medium at 30°C were either counted or shifted at 10°C for 24 h before counting. For each condition tested, 300 to 400 cells from 2 independent clones were scored. (A) Large-budded, M, small-budded, S/G2, and unbudded, G1 cells were scored. Percent of cells in M phase was significantly different in SA and SE strains as compared to WT strain. *** *p*<0.001, χ^2^ test comparisons. (B) Nuclei of cells were stained with Hoechst. The percentages of four types of nuclear morphology in large-budded mitotic cells are indicated: an undivided nucleus in one cell body, an undivided nucleus at the bud neck, divided nuclei properly segregated into each cell body, and divided nuclei both located in one cell body. Distributions were significantly different in SA and SE cells as compared to WT cells: *p*<0.001 using χ^2^ test comparisons.

We then assayed which phase of the cell cycle was affected in the growth defect at 10°C of mutant SE strain. Large-budded (mitotic phase), small-budded (S/G2 phase) and unbudded (G1 phase) cells were counted after 24 hour at 30°C or 10°C. At 10°C, SA and SE strains exhibited an accumulation of cells with a large bud (Figure 2A). Although SA strain was able to grow better than WT cells at 10°C (Figure 1C), we observed a two-fold increase of large-budded mitotic cells as compared to WT strain (32.1% vs. 16.2%, respectively, Figure 2A). Therefore, the accumulation of mitotic cells for SA clones at 10°C did not impair growth capacities of these clones. Accumulation of large-budded cells was much more dramatic for SE strain (57.4%, Figure 2A), and was concomitant with a strong impairment of growth at 10°C (Figure 1C). This result suggested a mitotic block for SE cells at 10°C.

### Defects in nuclear positioning and segregation during mitotic phase

An accumulation of cells in mitotic phase could be correlated with a default in nuclear positioning and/or a default in nuclear division. Indeed, mitotic spindle must be correctly positioned at the bud neck for anaphase onset to occur. Therefore, we counted, for the different strains, nuclei number and position in large-budded cells at 10°C (Figure 2B). An abnormal mitotic feature with an undivided nucleus positioned far from the bud neck in the mother cell was more frequent in SA and SE strains as compared to WT strain (8% and 21% vs. 3%, respectively). This feature for SA and SE cells could reflect a delay in anaphase onset due to abnormal nucleus positioning. Furthermore, in 12% of SA large-budded cells, two divided nuclei in the mother cell were observed. Hence, in these cells, anaphase occurred, but in an incorrect manner. This might reflect again a problem with the nucleus positioning machinery.

Additionally, in large-budded SA and SE cells we observed a general reduction in the number of cells with divided nuclei as compared to WT cells (21+12 = 33% and 17+2 = 19% vs. 53+1 = 54%, respectively, Figure 2B), which may reflect defective intra-nuclear spindle MT function.

### Nuclear oscillations are impaired in pre-anaphase SA and SE cells

**Figure 3:**
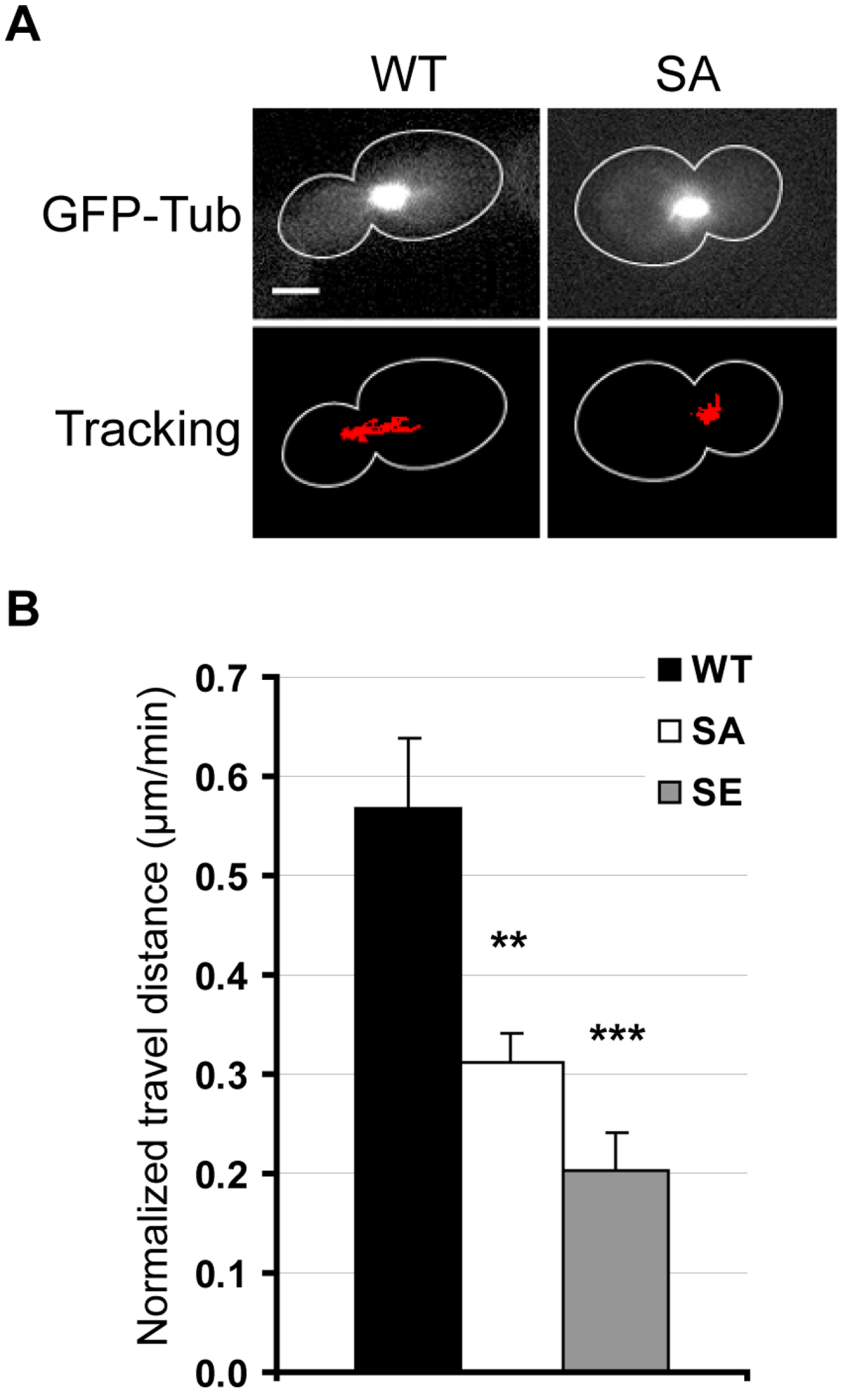
Nuclear movements are inhibited in SA and SE cells prior to anaphase. WT, SA and SE cells were transformed with a plasmid expressing GFP-Tub1p, and were analyzed by time-lapse microscopy. (A) Representative WT and SA pre-anaphase cells for which the bud-directed SPB was tracked at each time point of the time-lapse experiment. The track is overlaid in red on an image of the movie. Scale bar, 3 µm. Movies available in supplementary Figure S1. (B) Measurement of the travel distance covered by the bud-directed SPB in pre-anaphase cells over a period of 15–30 min (results are normalized in µm/min). Comparing to WT strain, spindle movements were reduced in both mutant strains. Number of analyzed cells: WT, n = 19; SA, n = 15; SE, n = 17. Error bars are SEM. ** *p*<0.01, *** *p*<0.001, *t* test comparisons of mutant cells vs. WT cells.

The nucleus positioning machinery in pre-anaphase cells depends on cytoplasmic MTs and involves nucleus oscillations [18]. To observe these oscillations, WT, SA and SE cells were transformed with a *GFP-TUB1* construct, to label the pre-anaphase intra-nuclear spindle in live cells (Figure 3A). Using time-lapse microscopy, we could observe that the movement of pre-anaphase spindle was reduced in SA (Figure 3A and supplemental Video S1) and in SE strains (not shown). The travel distance covered by the bud-directed SPB in pre-anaphase cells was measured over a period of 15 to 30 min and results were normalized in µm/min (Figure 3B). Results show a reduction of approximately 50% in both mutant strains and clearly indicate a defect in nuclear oscillations, most probably linked to defective cMT functions.

### Altered nucleation and/or elongation activities of cMTs in preanaphase SA and SE cells

**Figure 4:**
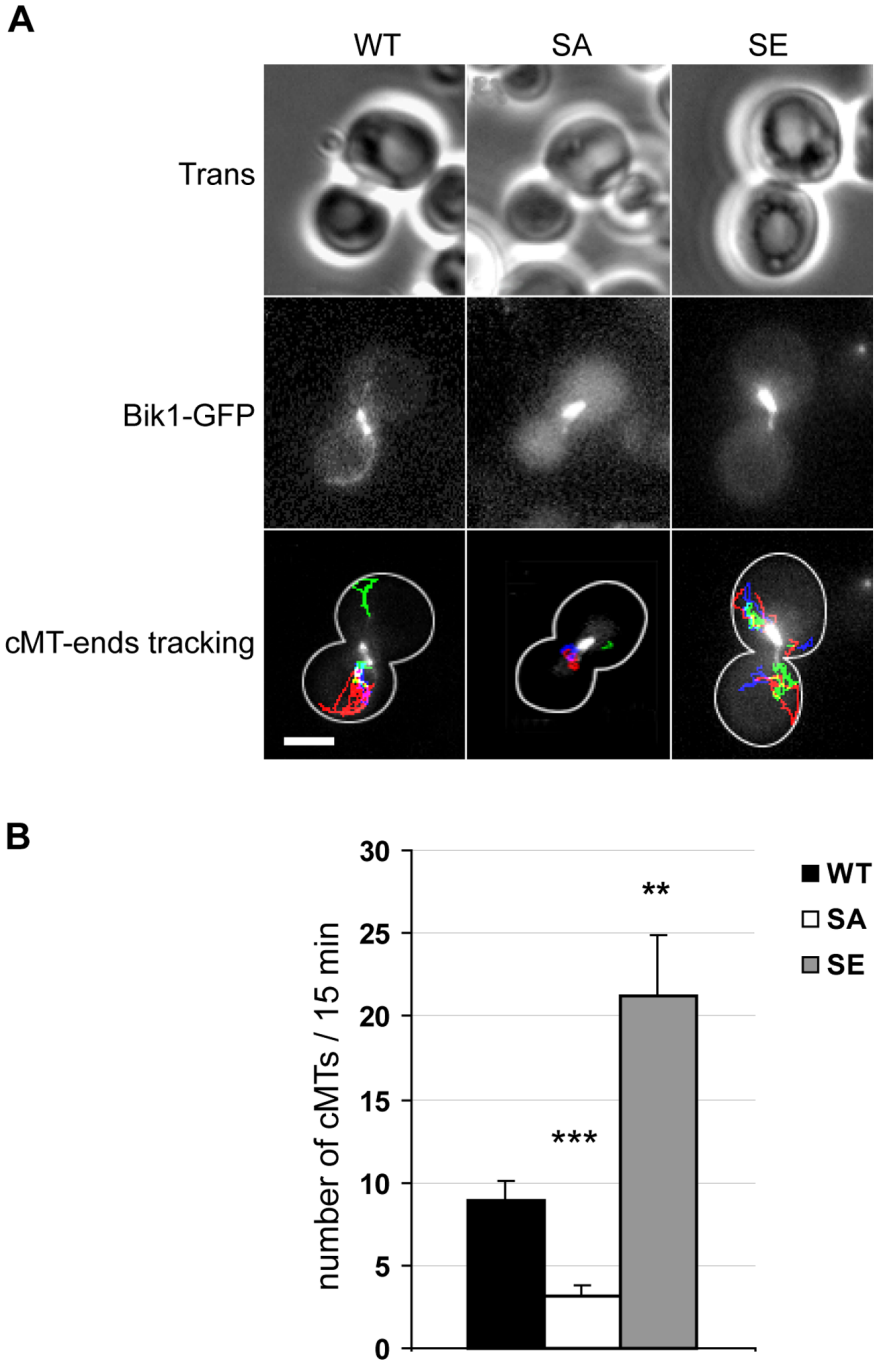
Nucleation and/or elongation activities of cMTs are modified in SA and SE cells. WT, SA and SE cells were transformed with a plasmid expressing Bik1p-GFP, and were analyzed by time-lapse microscopy. (A) Representative WT, SA and SE pre-anaphase cells for which the plus-end of each cMT was tracked at each time point of the time-lapse experiment. Tracks are overlaid on an image of the movie. Scale bar, 3 µm. Movies are available in supplementary Figure S2. (B) The number of cMTs that appeared in 15 min in pre-anaphase cells was counted. Comparing to WT cells, this number was reduced in SA cells, but was greatly increased in SE cells. Number of analyzed cells: WT, n = 10; SA, n = 11; SE, n = 11; error bars are SEM. ** *p*<0.01, *** *p*<0.001, *t* test comparisons of mutant cells vs. WT cells.

In order to visualize cMTs in living cells, the strains were transformed with a *BIK1-GFP* construct. We observed an overall reduction of the number of cMTs in pre-anaphase SA cells as compared to WT cells or SE cells. To get a measure of this phenotype, we scored the number of cMTs appearing during a period of 15 min in SA, SE and WT cells (Figure 4 and supplemental Video S2). This number was dramatically reduced in SA cells whereas it was greatly enhanced in SE cells as compared to WT cells (3.2±0.6 and 21.2±3.7 vs. 8.9±1.2 cMTs, respectively). These results indicate that both mutants present altered nucleation and/or elongation of MT seeds.

### Cytoplasmic MTs in SA and SE mutants exhibit altered dynamics

**Figure 5:**
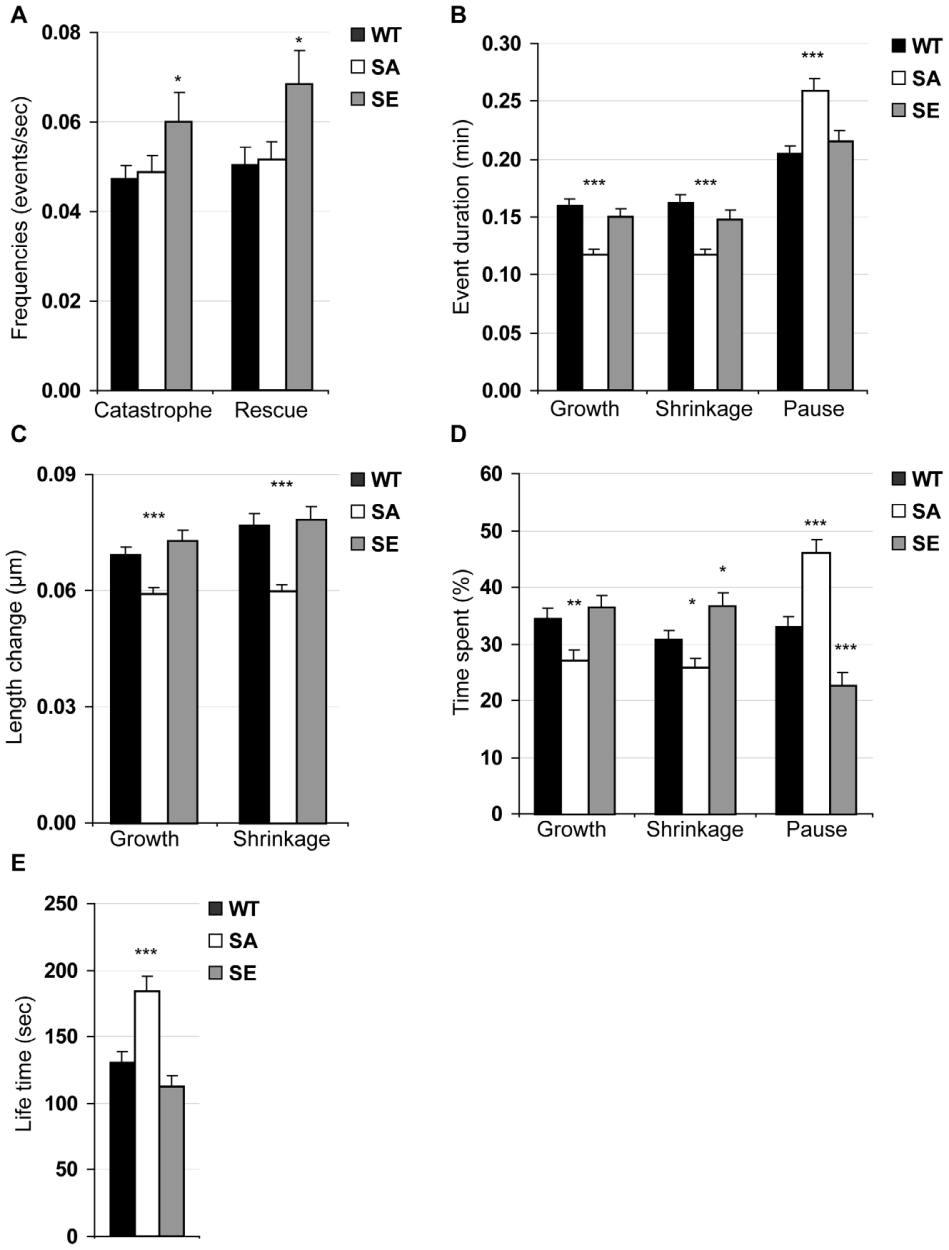
Bud-directed cMT dynamics in WT, SA and SE cells. Values are taken from Table 1 and only data for which significant differences between a mutant strain and the WT strain were found were represented here. (A) Frequencies of catastrophes and rescues, (B) growth, shrinkage and pause mean durations, (C) mean length change during growth and shrinkage, (D) total time spent while growing, shrinking and pausing, and (E) mean life time. Comparing to WT cells, many parameters of cMT dynamics varied in mutant cells, with SA cMTs behaving very differently from SE cMTs. Error bars are SEM. * *p*<0.05, ** *p*<0.01, *** *p*<0.001, *t* test comparisons of mutant cells vs. WT cells.

In order to measure bud-directed cMT dynamic parameters, *BIK1-3GFP* and *GFP-TUB1* were integrated in WT, SA and SE strains and time-lapse microscopy was performed. Results are presented in Table 1 and significant differences between strains in Figure 5.

**Table 1:** Dynamics of bud-directed cMTs in pre-anaphase cells.

	WT	SA	SE
Growth rate (µm/min)	0.56±0.02 (425)	0.63±0.02 (307)	0.61±0.02 (277)
Shrinkage rate (µm/min)	0.57±0.03 (380)	0.63±0.04 (284)	0.65±0.05 (273)
Catastrophe frequency (events/sec)	0.0472±0.0031 (354)	0.0487± 0.0039 (272)	0.0599±0.0065 (247)
Rescue frequency (events/sec)	0.0505±0.0040 (375)	0.0516±0.0040 (292)	0.0686±0.0072 (239)
Growth duration (min)	0.16±0.006 (425)	0.12±0.004 (307)	0.15±0.006 (277)
Shrinkage duration (min)	0.16±0.008 (380)	0.12±0.005 (284)	0.15±0.008 (273)
Pause duration (min)	0.20±0.007 (328)	0.26±0.012 (228)	0.22±0.010 (178)
Length change during growth (µm)	0.070±0.002 (425)	0.059±0.001 (307)	0.073±0.002 (277)
Length change during shrinkage (µm)	0.077±0.003 (380)	0.060±0.002 (284)	0.078±0.003 (273)
Time spent in growth (%)	35±1.7 (425)	27±1.6 (307)	36±2.1 (277)
Time spent in shrinkage (%)	31±1.5 (380)	26±1.6 (284)	37±2.2 (273)
Time spent in pause (%)	33±1.8 (328)	46±2.4 (228)	23±2.3 (178)
Life time (sec)	131±8 (94)	184±12 (43)	112±9 (68)

Time lapse sequences lasting a total of 11,945 sec for WT cells; 7,763 sec for SA cells; and 7,375 sec for SE cells were analyzed. Results are average values ± SEM. Values in parentheses are the number of events, except for life time for which values in parentheses are the number of MTs.

When comparing SA cells with WT cells, we observed significant differences in event durations: growth and shrinkage mean durations were lower in SA cells, but pause mean duration was higher (Figure 5B). Consistently, SA cMTs spent a reduced total time in growing or shrinking and an increased total time in pausing as compared to WT cMTs (Figure 5D). As a consequence, length changes during growth or shrinkage were reduced in SA strain (Figure 5C), and cMT mean life time was augmented (Figure 5E). Hence, cMTs in SA cells are less dynamic.

In SE cells, mean durations of growth, shrinkage and pause were very similar to those observed in WT cells (Figure 5B). However, total time spent pausing by cMTs was lower in SE cells than in WT cells, while time spent shrinking was augmented in SE cells (Figure 5D). Also, catastrophe and rescue frequencies were enhanced in SE cells (Figure 5A). Hence, cMTs in pre-anaphase SE cells are more dynamic than cMTs in WT cells.

In conclusion, results presented in Figures 4 and 5 show altered properties of bud-directed cMTs in both SE and SA mutant strains: SA mutation reduces the number and dynamics of cMTs whereas the phospho-mimetic SE mutation increases both the number and dynamics of cMTs. Altogether, these results indicate that normal dynamics of cMTs in yeast cells rely on the β tubulin Ser172 residue, which might be regulated by phosphorylation. As cMT dynamics are tightly controlled by MT effectors, it is worth testing the functionality of MT effectors in mutant strains.

### Ser172 mutations impair normal function of microtubule effectors

In order to test MT effectors, we chose to investigate synthetic lethal interactions of WT, SA or SE strains with strains deleted in *DYN1*, *BIK1*, *BIM1*, *KAR9* or *KAR3*. Bim1p and Kar9p belong to the Kar9p pathway for pre-anaphase spindle positioning at the bud neck, while Dyn1p and Bik1p belong to the Dyn1p pathway. Besides, Bik1p, Bim1p, and the minus-end directed motor Kar3p have been shown to have important functions in the regulation of spindle MTs [24], [25], [26], [27].

**Figure 6:**
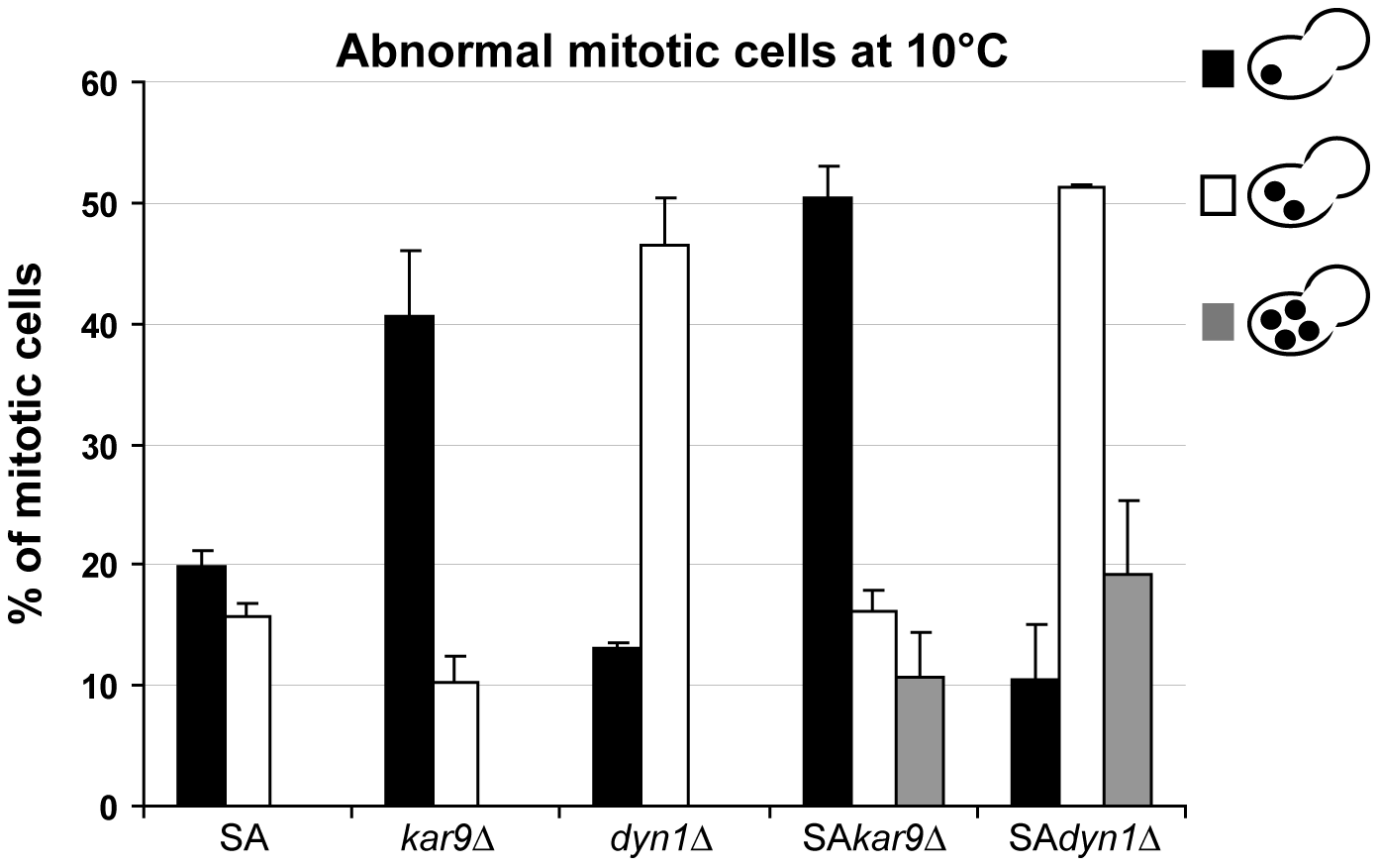
SA mutation worsens *kar9Δ* or *dyn1Δ* deletion phenotypes, with a number of double-mutant mitotic cells exhibiting polyploidy. SA, *kar9Δ*, *dyn1Δ* simple mutant cells or SA/*kar9Δ* and SA/*dyn1Δ* double mutant cells, were grown in liquid medium overnight at 30°C and shifted for 24 h at 10°C. Cells were then fixed, nuclei stained with Hoechst and nuclear number and position in large-budded mitotic cells were scored. Results are presented as percentages of a total of 400 mitotic cells from 2 independent clones, for each condition tested. Only abnormal mitotic patterns are shown in the graph. Error bars are SEM.

One remarkable result of these synthetic lethal interactions is that neither the SA nor the SE mutation is lethal when combined with *dyn1Δ* or *kar9Δ* deletions (Table 2). Thus, the observed defects of SA and SE strains in nuclear positioning and oscillations (Figures 2 and 3) are probably based on a partial alteration of both Dyn1p and Kar9p pathways. In this case, SA or SE mutation should worsen *KAR9* or *DYN1* deletion phenotypes. We thus analyzed mitotic features of SA, *kar9Δ*, *dyn1Δ*, SA/*kar9Δ* and SA/*dyn1Δ* strains at 10°C (Figure 6). Mitotic SA, *kar9Δ* and *dyn1Δ* cells exhibited abnormal features with either a nucleus improperly located (20%, 41%, 13%, respectively) or two nuclei in the mother cell (16%, 10% and 47%, respectively). Furthermore, the SA mutation worsened the *kar9Δ* phenotype with the apparition of polyploid cells (11%) in SA/*kar9Δ* double mutant strain. In double mutants SA/*dyn1Δ*, mitotic polyploid cells (19%) were also observed (Figure 6).

**Table 2:** Synthetic lethal interactions.

	WT			SA			SE		
	predicted	obtained	viable	predicted	obtained	viable	predicted	obtained	viable
*kar9Δ*	11	10	Yes	10	10	Yes	11	8	Yes
*dyn1Δ*	11	11	Yes	10	10	Yes	14	12	Yes
*bik1Δ*	15	14	Yes	13	0	No	7	0	No
*bim1Δ*	10	9	Yes	8	0	No	8	1	No
*kar3Δ*	9	7	Yes	11	1	No	10	0	No
*mad2Δ*	25	25	Yes	41	36 (31 small)	Sick	45	44 (25 small)	Sick

WT, SA or SE haploid cells were crossed with *dyn1Δ*, *bik1Δ*, *kar9Δ*, *bim1Δ*, *kar3Δ* and *mad2Δ* haploid cells. Crossed diploid cells were sporulated and dissected to assess the viability of resulting haploid double-mutant spores. Double-mutant spores viable and forming a colony was counted as “obtained”, and counts of “obtained” were compared to “predicted” double-mutant spores. Results are: Viable (Yes) when “obtained” was equal or nearly equal to “predicted”; Synthetic Lethal (No) when no “predicted” double-mutant colony was “obtained”, and Synthetic Sick (Sick) when most obtained double-mutant colonies are of small size.

In conclusion, double mutants SA/*dyn1Δ* and SA/*kar9Δ* exhibited many errors in nuclear positioning and nuclear segregation, with the apparition of polyploid cells. Thus, Ser172 residue in β tubulin appears important for a normal function of Dyn1p and Kar9p pathways at the mitotic stage in yeast cells.

Additionally and very interestingly, SA and SE mutations which are not synthetic lethal with deletion of *KAR9* or *DYN1* are synthetic lethal with deletions of other components of the two pathways, namely *BIK1* and *BIM1* (Table 2). Thus, another function of Bik1p and Bim1p than spindle positioning is responsible for this lethality. Moreover, synthetic lethality is also observed with *Kar3Δ* deletion. Because the three proteins, Bik1p, Bim1p and Kar3p, are known to act on kinetochores and interpolar MTs [24], [25], [26], [27], the observed synthetic lethality with SA and SE mutations indicate that β tubulin Ser172 may have a vital function inside the spindle.

To check the integrity of the mitotic spindle in mutant strains, SA, SE and WT cells were mated with *MAD2* deleted cells. Mad2p is a component of the spindle assembly checkpoint [28]. When it is deleted, cells can bypass the spindle assembly checkpoint and enter anaphase. If the spindle is normal, the bypass of the checkpoint has no consequence on cell viability. But, if the spindle exhibit defaults, the bypass of the checkpoint induces an accumulation of damages at each cell cycle, impairing overall cell viability. We found that SA and SE cells were synthetic sick with the deletion of *MAD2* (Table 2). Therefore, the two mutations SA and SE affect the spindle, and, in the absence of the checkpoint, give rise to small colonies.

## Discussion

We showed previously in mammalian cells that β tubulin Ser172 is phosphorylated during mitosis by the cyclin-dependent kinase Cdk1 [21]. The aim of the present study was to challenge the importance of the β tubulin Ser172 residue in cell physiology using the yeast *S. cerevisiae*. Although Ser172 phosphorylation in yeast has not been detected yet, we demonstrate that mutation of the Ser172 residue (replaced either by an Alanine or a Glutamic Acid) has profound effects on microtubule properties and on cell cycle parameters.

At 30°C, yeast mutant cells are viable and exhibit both intranuclear and cytoplasmic MTs demonstrating their ability to assemble mutant SA or SE β tubulin. Such result was surprising in the case of the SE mutant, because mammalian cells expressing a mutant S172D or S172E β tubulin were only poorly able to incorporate this mutant tubulin in their MTs [21]. The major difference between the two studies is that yeast mutant cells express *tub2*-S172E as their unique source of β tubulin, while in mammalian cells, the mutant S172E β tubulin was expressed among normal β tubulin isoforms. The SE yeast strain was supersensitive to benomyl and was cold-sensitive. Therefore in yeast, MTs could incorporate *tub2*-S172E β tubulin but they most probably present structural alterations.

Cytoplasmic MTs of SA and SE mutant cells, at 10°C, show disturbed functions: SE strain has a large number of abnormal mitotic cells with a nucleus improperly located away from the mother-bud neck, and many SA mitotic cells show two nuclei in the mother cell. Thus, in the two mutants, the nucleus positioning machinery is impaired as also confirmed by the observed inhibition of nuclear oscillations. Mechanistically, the nucleus positioning machinery relies on two spindle positioning pathways before anaphase: the Kar9p pathway (Kar9p/Bim1p/Myo2p) and the dynein pathway (Dyn1p/Bik1p/Kip2p). Our synthetic interaction studies suggest that SA and SE mutations affect partially both dynein and Kar9p pathways, maybe by affecting the association of cMTs with specific effectors of each pathway. Effectors of both pathways are targeted by the Cdk1/Cdc28p kinase. In complex with the mitotic cyclins Clb4p and Clb5p, Cdc28p phosphorylates Kar9p, conferring tight spatial control of Kar9p to the bud-cell-bound SPB, and promoting asymmetric Kar9p transport to cMT plus-ends in the bud [29], [30], [31], [32]. Also, the late mitotic cyclins Clb1p and Clb2p regulate the preferential Dyn1p distribution to bud-directed SPB and cMTs [33]. Hence, Cdk1/Cdc28p seems to play a very general role in the spatial control of mitotic events. A putative phosphorylation of β tubulin on Ser172 by Cdk1/Cdc28p might be part of this control.

Intranuclear spindle MTs are also affected in SA and SE mutants. At 10°C, mutant strains exhibit a low proportion of mitotic cells with divided nuclei, indicating that anaphase does not readily occur. Furthermore, deletion of *BIK1*, *BIM1* or *KAR3* in SA or SE cells is lethal. Bik1p, Bim1p and Kar3p have important roles in regulating kinetochore MTs and interpolar MTs in the spindle [24], [25], [26], [27] and the Ser172 mutations appears to be detrimental to this function. Corroborating the view that spindle function is abnormal in mutant cells, SA and SE mutants become synthetic sick when mated with cells deleted for Mad2p, an effector of the spindle assembly checkpoint. It is known that interference with MT dynamics activates the checkpoint [28]. In SA and SE cells, MT dynamics are altered and, as expected, the checkpoint appears to be active, as revealed by the deleterious effect of *MAD2* deletion.

In yeast cells, the effects of two other β tubulin mutations surrounding the Ser172 residue have been studied. The first one, *tub2-429*, is a double mutation *tub2*-K174A/D177A [23], where K174A is located within the putative S^172^PK phosphorylation consensus site. The *tub2-429* mutant shares common traits with S172 tubulin SA and SE mutants: i) it is viable at normal growth temperature but exhibits benomyl-supersensitivity and cold-sensitivity and ii) at 12°C, it is arrested in its cell cycle as large-budded cells with undivided nuclei [23]. The second mutation very close to Ser172 was on Val 169, which is supposed to interact with GTP [34]. The *tub2*-V169A strain exhibits substantial changes in both cytoplasmic and kinetochore MT dynamics. Furthermore, this strain grows slowly, and pre-anaphase cells contain a high proportion of monopolar chromosomes attached to only one spindle pole [34]. In any case, it seems that the region surrounding Ser172 is crucial for the MT functions via GTP binding, tubulin conformation [22] or tubulin phosphorylation [21].

In conclusion, our model mutants showed that the Ser172 site, phosphorylated or not, is of primary importance for spindle machinery and cell mitosis.

## Materials and Methods

### Yeast strains and plasmids

The *S. cerevisiae* strains and plasmids used in this study are listed in Table 3. The S172A (SA) and S172E (SE) mutations were obtained by directed mutagenesis on the *TUB2* gene carried by the p*RR190* plasmid [23], using the QuickChange kit (Stratagene, La Jolla, CA) and according to manufacturer's instructions. Codon replacement were TCT172GCT for SA mutation and TCT172GAA for SE mutation. For WT control, intact p*RR190* was used. WT, SA and SE haploid yeast strains were obtained as described in [23]. Briefly, p*RR190* plasmid or mutagenized p*RR190* plasmid was linearized and integrated in the CUY409 diploid strain at the *tub2-Δ1::LEU2/TUB2* locus. Ura+Leu- diploid transformants were then sporulated and dissected to obtain WT, SA or SE haploid Ura+ cells. The sequence of integrated *TUB2* (WT) or *tub2* (SA or SE) genes was checked after PCR amplification of a 1.8 kb genomic DNA fragment containing the *TUB2* gene (primers used for PCR amplification were as in [23]). Homozygous diploid strains were obtained by crossing haploid strains of identical genotype.

**Table 3:** Strains and plasmids used in this study.

yeast strains	genotype	source
CUY409	*MATa/alpha, ACT1:HIS3/ACT1, tub2-Δ1::LEU2/TUB2, his3-Δ200/his3-Δ200, ura3-52/ura3-52, lys2-801/lys2-801, ade2-101/ade2-101, leu2-Δ1/leu2-Δ1, Gal*+	Ref [23]
WT	*MATa, TUB2:URA3, his3-Δ200, ura3-52, lys2-801, ade2-101, leu2-Δ1, Gal*+	this study
SA	WT except *tub2-S172A:URA3*	this study
SE	WT except *tub2-S172E:URA3*	this study
WT *BIK1-3GFP GFP-TUB1*	WT except *BIK1-3GFP-HphR, GFP-TUB1-URA3*	this study
SA *BIK1-3GFP GFP-TUB1*	SA except *BIK1-3GFP-HphR, GFP-TUB1-URA3*	this study
SE *BIK1-3GFP GFP-TUB1*	SE except *BIK1-3GFP-HphR, GFP-TUB1-URA3*	this study
*dyn1Δ (YKR054c)*	BY4742, *MATalpha, his3Δ1, leu2Δ0, lys2Δ0, ura3Δ0, YKR054c::kanMX4*	euroscarf
*bik1Δ (YCL029c)*	BY4742, *MATalpha, his3Δ1, leu2Δ0, lys2Δ0, ura3Δ0, YCL029c::kanMX4*	euroscarf
*kar9Δ (YPL269w)*	BY4742, *MATalpha, his3Δ1, leu2Δ0, lys2Δ0, ura3Δ0, YPL269w::kanMX4*	euroscarf
*bim1Δ (YER016w)*	BY4742, *MATalpha, his3Δ1, leu2Δ0, lys2Δ0, ura3Δ0, YER016w::kanMX4*	euroscarf
*kar3Δ (YPR141c)*	BY4742, *MATalpha, his3Δ1, leu2Δ0, lys2Δ0, ura3Δ0, YPR141c::kanMX4*	euroscarf
*Mad2Δ YJL030w)*	BY4742, *MATalpha, his3Δ1, leu2Δ0, lys2Δ0, ura3Δ0, YJL030w::kanMX4*	euroscarf
**Plasmids**		
p*RR90*	*TUB2::URA3 AmpR*	Ref [23]
p*FC1*	*BIK1-3GFP-hphMX4 AmpR*	Ref [38]
p*B532*	*BIK1-GFP-LEU2 2µ AmpR*	Ref [24]
p*CJB1*	*GFP-TUB1-LEU2 AmpR (*backbone p*RS315)*	this study
p*AFS125*	*GFP-TUB1-URA3 AmpR*	Ref [35]

WT, SA and SE cells with integrated *BIK1-3GFP* and *GFP-TUB1* were obtained as follow: *TUB2-HIS3* haploid cells issued from the sporulation of CUY409 diploid strain, were transformed with integrating p*AFS125* [35]. Ura+His+ transformants were then crossed with WT, SA or SE haploid cells containing integrated *BIK1-3GFP* (p*FC1*), and diploids were sporulated and dissected. Non parental Ura+ ditypes were selected and checked by fluorescence for Bik1p-3GFP and GFP-Tub1p expression. Sequence of *TUB2*, *tub2*-S172A and *tub2*-S172E in these clones was verified by PCR as above.

### Cell growth and cytological analyses

For growth tests on plates, fresh overnight cultures of WT, SA and SE cells (2 different haploid clones for each strain) were tested for their OD_600_ and serial dilutions were spotted on YPD plates containing, or not, 15 µg/mL benomyl. Plates were incubated for 2 days at 30°C. For evaluation of cell growth in liquid medium, fresh cultures were diluted to OD_600_  = 0.1, and cultured for several hours at 30°C or 37°C, or several days at 10°C. Every growth test was done with 2 independent clones. Cell morphologies were analyzed on diploid homozygous cells by counting different types of cells (unbudded, small-budded, and large-budded) under a light microscope. For small-budded cells, we counted cells with a bud size smaller to ¾ of mother size. For large-budded cells, we counted cells with a bud size equal or larger than ¾ of mother size. The positions and the number of nuclei in large-budded cells were determined by staining with Hoechst 33258 (Sigma) as described [36]. The same experiments were performed with haploid cells issued from the dissections of spores of the synthetic lethality studies (Figure 6).

### Synthetic lethal interactions

Haploid WT, SA or SE cells were crossed with *dyn1Δ*, *bik1Δ*, *kar9Δ*, *bim1Δ*, *kar3Δ* or *mad2Δ* deletion strains from Euroscarf (Table 3). Diploid cells were sporulated and tetrads were dissected with an Axiolab micromanipulator (Zeiss), using standard procedures [36]. After germination, spores were replica-plated on selective media to check for the growth of double-mutant spores (Table 2).

### Time-lapse microscopy, measurement of MT dynamics and image analysis

Time-lapse sequences were collected on living yeast cells kept at 30°C using a Zeiss Axiovert microscope equipped with a Coolsnap ES CCD camera (Ropper Scientific) and controlled by Metamorph software (Universal Imaging). Five focal planes separated by 0.5 µm were taken at every time point using a piezoelectric motor. To assess nuclear (spindle) movement, images of yeast cells expressing GFP-Tub1p were taken at 30 sec intervals for a total of 60 min, with an exposure time of 500 msec at each focal plane. To examine nucleation and/or elongation activity, images of yeast cells expressing Bik1p-GFP were taken at 10 sec intervals for a total of 15 min, with an exposure time of 500 msec at each focal plane. To measure parameters of dynamic instability, images of yeast cells with integrated *GFP-TUB1* and *BIK1-3GFP* were taken at 3.5 sec intervals for a total of 5–10 min, with an exposure time of 600 msec at each focal plane. Tracking of the nuclear position in the cell and tracking of cMTs was performed using Metamorph software. For MT dynamics measurements, MT length at each time point was measured manually on maximal projections and dynamics parameters were calculated as described in [37], using a home-made Visual Basic macro embedded in a Microsoft Excel datasheet (code available on request).

### Statistics

All statistics presented were performed using Prism 4.0 (GraphPad, San Diego, USA).

## Supporting Information

Video S1 Spindle movements are reduced in SA cells as compared to WT cells. WT and SA cells were transformed with a plasmid expressing GFP-Tub1p to label spindle MTs and were analyzed by time-lapse microscopy for 60 min at 30 sec intervals. Spindle movements through the bud neck were reduced in SA cells (right). Bar, 3 µm.
(1.58 MB MOV)

Video S2 Nucleation and/or elongation activities are reduced in SA cells and enhanced in SE cells. WT, SA and SE cells were transformed with a plasmid expressing Bik1p-GFP to label both spindle and cytoplasmic MTs, and were analyzed by time-lapse microscopy for 15 min at 10 sec intervals. Compared to WT cells (left), cytoplasmic MTs appearing in 15 min were less numerous in SA cells (middle) and more numerous in SE cells (right). Bar, 3 µm.
(2.06 MB MOV)
